# Performance of Low-Cost Agar from *Gracilaria salicornia* on Tissue Culture of *Pleurotus* HK-37

**DOI:** 10.1155/2019/2565692

**Published:** 2019-11-03

**Authors:** Farid Mzee Mpatani, Said Ali Hamad Vuai

**Affiliations:** Department of Chemistry, College of Natural and Mathematical Sciences, The University of Dodoma, P.O. Box 338, Dodoma, Tanzania

## Abstract

Currently, the demand of *Pleurotus* HK-37 (oyster mushroom) in Tanzania is growing rapidly due to the increasing of awareness on its nutrition, health, and economic benefits. Despite the increasing demand, the availability of strains of *Pleurotus* HK-37 species is still a challenge due to high cost of tissue culture technology. The high cost of importing agar seems to be among the factors for this failure. This study aimed at investigating the performance of low-cost agar from local *Gracilaria salicornia* on tissue culture of *Pleurotus* HK-37. Local extracted agars with different gel strengths ranging between 100, 200, 300, 400, and 500 g/cm^2^ were used to make PDA media. The average mycelia growth rate (mm/day) ranged between 9.87 ± 1.44 and 14.9 ± 0.85 mm/day. Low-cost agar shows quite similar performance as that of standard agar on active growth of *Pleurotus* HK-37 mycelia. All PDA plates appeared white and feathery and showed to grow in a circular mode (radial extension). Mycelia growth on standard agar PDA took 5 days while on extracted local agar PDA took 5 to 7 days to fully colonize the plate at 27 ± 2°C. The present study shows that the production cost can be reduced by ∼35–78% by using local agar.

## 1. Introduction

*Pleurotus* spp. (oyster mushroom) is one of the most common edible mushrooms produced in the world, and its cultivation has increased gradually all over the world [1]. This species is easily cultivated on various substrates and has high nutritional value with medical properties [2, 3]. *Pleurotus* HK-37 is among the oyster mushrooms grown in Tanzania originated from South Africa [4]. However, the strains of this species are still not performing well in Tanzania. Among the factors that associated with poor performances is low quality of strains as a result of using local propagation methods, lack of uniformity among strains, and disease facing spawns because of nonsterile cultivation conditions. Tissue culture methods have received much attention as a progressive technology of rapid regeneration and multiplication of mushroom spawns, as it play advantage of producing high quality and uniform spawns. These spawns can be prolonged and multiplied under disease-free conditions [5]. In Tanzania, this technology is costly since the technique depends entirely on importation of agar which keeps the agar costs high and subsequently increases the cost of the production for small and medium industries [6].

Agar is the most expensive substances in the media which contributes around 70% costs [7–9]. Kivaisi and Buriyo [10] explained on the high cost of using commercial agar for microbiological purposes in Tanzania. Studies are still ongoing seeking appropriate substrates to replace agar in culture medium due to the high cost of grade agar [11, 12]. Several substrates have been used as substitutes for agar; these include alginates [13], starch [14, 15], isubgol [16], and xanthan gum [17]. Nonetheless, the performance of most of the substrates are not of certain as they possess poor gelling ability which results in softening of media and need additional ions for effective gelling and rapid dispensing [17]. Looking for production of low-cost agar from Tanzania's seaweed is necessary to overcome the challenges. Therefore, the present work aimed at establishing the techno-economic production of low-cost high-grade agar from the local seaweed species *Gracilaria salicornia* and assessing its performance in tissue culture of *Pleurotus* HK-37. Progressing on tissue culture technique can boost utilization of *Pleurotus* HK-37 and other mushroom species in Tanzania. Tanzania's coast is endowed with abundant red algae seaweeds include *Gracilaria salicornia* [18]. Few studies done have shown the potential of using agar extracted from local *Gracilaria salicornia*. However, recently, no study has been done on evaluating the production cost of agar from local *Gracilaria salicornia* and investigating its application in tissue culture of *Pleurotus* HK-37.

## 2. Materials and Methods

### 2.1. Materials

**Figure 1:**
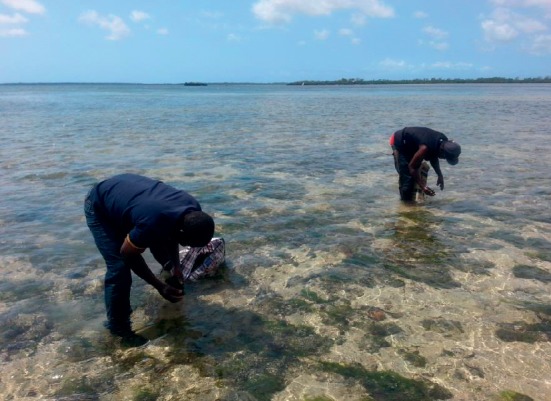
Collection of *Gracilaria salicornia* (green patches) at Chwaka Bay, Zanzibar, Tanzania.

Samples of *Gracilaria salicornia* for production of agar were collected at Chwaka Bay in Zanzibar, Tanzania (Figure 1). *Pleurotus* HK-37 was taken from the Department of Molecular Biology and Biotechnology, University of Dar es Salaam (UDSM), Tanzania. White flesh, red-skinned potatoes (*Solanum tuberosum* L.) were used in potato dextrose agar (PDA) media preparation and sorghum (*Sorghum bicolor*) grains were used for *Pleurotus* HK-37 spawn preparation. These food materials were bought from Darajani Market in Zanzibar, Tanzania. The tissue culture of *Pleurotus* HK-37 was done in microbiological unit at the Chief Government Chemist Laboratory Agency (CGCLA), Zanzibar, Tanzania.

### 2.2. Experimental Design

Alkali treatment of *Gracilaria salicornia*, agar extraction, and determination of gel strength, sulfate content, gelling temperature, and melting temperature were done as reported in [6]. Extracted local agars from alkali-treated and nontreated samples with different gelling strength ranging between 100, 200, 300, 400, and 500 g/cm^2^ were obtained from the study of [6]. Tissue culture of *Pleurotus* HK-37 was done with minor modification according to the methods described in [1, 19]. The PDA medium was prepared using 1.5% (w/v) agar (nontreated and alkali-treated, each separately) on plates according to [20]. Oxoid agar powder, bacteriological, as a standard was used for comparison. Brief description of the method used is outlined below.

### 2.3. Preparation of Agar Media

Five PDA media were prepared using local agars extracted at different parameters as shown in Table 1; namely, nontreated agar (120°C and 2 h), treated agar (10% NaOH; 0.5 h and 115°C), treated agar (20% NaOH; 2 h and 120°C), treated agar (30% NaOH; 2 h and 115°C), and treated agar (30% NaOH; 2 h and 120°C). A portion of 200 g of sliced, unpeeled potatoes was boiled in 1000 mL of distilled water for 30 min to prepare PDA media. The solution was filtered through cheesecloth to get potato infusion which is equivalent to 4.0 g of potato extract. The infusion was mixed with 20 g of dextrose anhydrous, 15 g of extracted agar, and water up to the maximum of 1000 mL and then boiled to dissolve. The solution was autoclaved at 121°C for 15 min. The medium was later acidified to pH 3.5 at 50°C using 10% tartaric acid. The medium was mixed well before pouring it in PDA plates with 90 mm diameter. The same procedures were followed to prepare PDA using oxoid agar powder, bacteriological.

### 2.4. Preparation of Mycelia Growing Culture from *Pleurotus* HK-37 Strain

**Figure 2:**
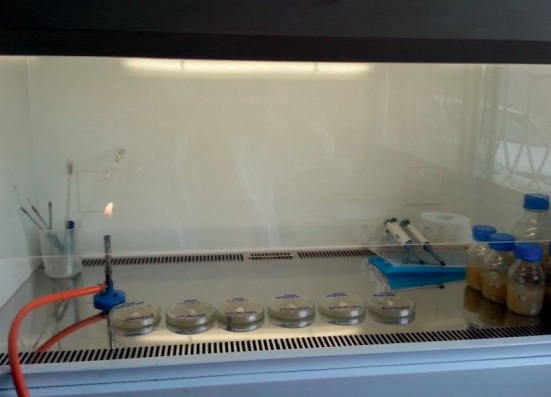
Strain of *Pleurotus* HK-37 (white patch) aseptically transferred to the center of each PDA media.

In a laminar flow cabinet, a scalpel was flamed until red-hot and then cooled in agar media-filled PDA plate. A small fragment of *Pleurotus* HK-37 strain (1 cm^2^) was cut using sterile cork borer and removed by a flame-sterilized scalpel. Quickly the fragment was aseptically transferred to the center of the nutrient-filled PDA plate (Figure 2). The PDA media plates were labeled with the date and kind of agar medium filled on. The plates (three replicates for each PDA medium) were incubated at 27 ± 2°C and monitored for the growth of mycelia.

### 2.5. Mycelia Growth Rate

The diameter of the colony in each plate was measured in millimeters as described in [21] for every 24 h using transparent ruler across the plate. The days took for mycelia to completely colonize the medium was noted. The growth rate of *Pleurotus* HK-37 was obtained by measuring the colonies diameter per day.

### 2.6. Substrate Preparation for Growth of Spawn

*Pleurotus* HK-37 spawn was grown on sorghum grains. 120 g of sorghum grains was thoroughly washed four times in sufficient water to remove unwanted particles and then put in a container soaked with water for 1 h. The grains were put in quart Mason jar and added on it three-quarter distilled water and then boiled until grains became semisoft. Excess water from the boiled grains was removed by spreading on sieve made of cotton cloth. The grains were left on the sieve to evaporate water. The grains were then mixed with 0.6 g of chalk powder (calcium carbonate) and 2.4 g of gypsum (calcium sulfate) to adjust pH and prevent sticking, respectively. Gypsum and chalk powder were first mixed separately and then thoroughly mixed with the grains. The prepared substrate filled in quart Mason jar was sealed with cotton and masked with aluminum foil. The jar was then sterilized at 121°C for 15 min. The sterilized jar was left in the room for cooling and kept on laminar flow under UV tube for 20 min before inoculation.

### 2.7. Inoculation of Sterilized Grain from Agar Media and Preparation of Mother Spawn

The mycelium-covered agar in growing culture was cut into three mycelia agar fragments (1 cm^2^) using sterile cork borer, removed by a flame-sterilized scalpel, and aseptically transferred to a quart Mason jar containing sterilized grains. An inoculated jar was agitated to dispense the mycelia and incubated in darkness at 27 ± 2°C. This was done separately for each agar PDA covered mycelia.

### 2.8. Cost Analysis

Cost analysis was done to evaluate the costs of the production of 500 g agar in each of five selected local agars having gel strengths in categories of 100, 200, 300, 400, and 500 g/cm^2^. The costs of agar production were assessed by considering operating cost, sample (*Gracilaria salicornia*) cost, chemicals and reagents cost, power (electricity) consumption cost, and fixed cost during agar extraction. These costs were compared with the market price of 500 g of Oxoid agar powder, bacteriological. *Gracilaria salicornia* samples were collected free without any charge.

### 2.9. Statistical Analysis

Data were analyzed by one-way analysis of variance (ANOVA), *P*=0.05. Pearson product-moment correlation coefficient in IBM SPSS Statistics 20 was used to measure the correlations and interactions of variables of local agar on growth of Pleurotus HK-37. The values of data were presented as mean ± SD (*n* = 3). All experiments were performed in triplicates.

## 3. Results

### 3.1. Physicochemical Characteristics and Cost of Agar

The physicochemical characteristics of agar, production cost, and mycelia growth rate (mm/day) of *Pleurotus* HK-37 on PDA media at 27 ± 2°C are determined as presented in Table 1.

### 3.2. *Pleurotus* HK-37 Mycelia Growth Rate and Formation of Spawns

**Figure 3:**
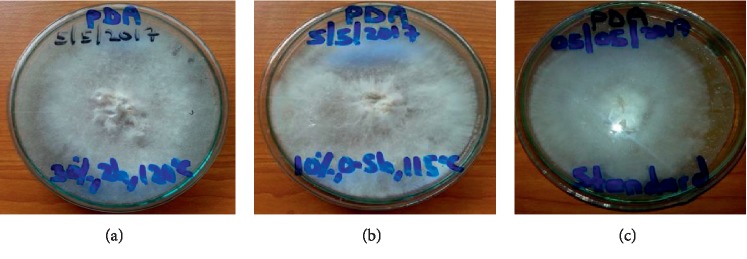
Pleurotus HK-37 mycelia covered on PDA plates of extracted agars (a), (b), and a standard-bacteriological agar (c).

**Figure 4:**
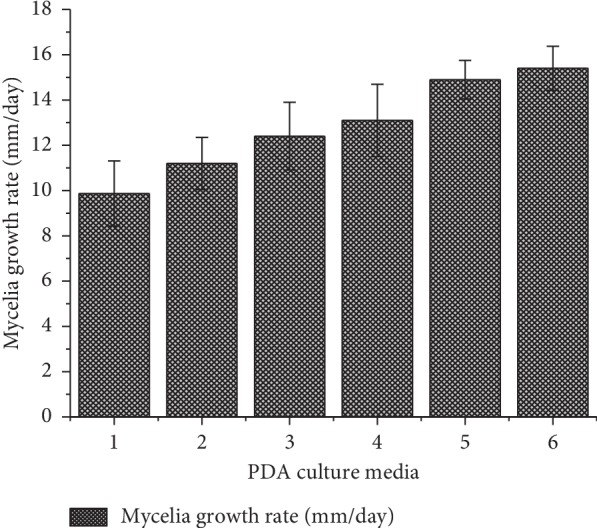
Observed mycelia growth rate of *Pleurotus* HK-37 at 27 ± 2°C on six different agar PDA culture media.

**Figure 5:**
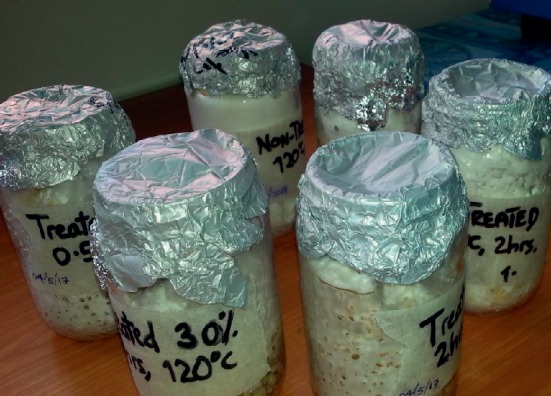
Pleurotus HK-37 colonized in sterilized jars filled with sorghum grains.

The mycelia of *Pleurotus* HK-37 in all plates appeared white and feathery and showed to grow in a circular mode (radial extension) as seen in Figure 3. The radial extension rates were equivalent to the growth rate of the mycelia. The mean mycelia growth diameter was obtained from three replicates of each agar PDA media investigated. The results showed that the mycelium of *Pleurotus* HK-37 took 5, 6, and 7 days for complete growth in PDA media at 27 ± 2°C. The average mycelia growth rate on media ranged between 9.87 ± 1.44 and 15.4 ± 0.98 mm/day with the highest and lowest obtained from standard PDA and nontreated PDA, respectively. The plates of PDA media of standard agar and treated agar of (30% NaOH; 2 h and 120°C) took 5 days, treated agar of (30% NaOH; 2 h and 115°C) and treated agar of (20% NaOH; 2 h and 120°C) took 6 days, and treated agar of (10% NaOH; 0.5 h and 115°C) and nontreated agar (120°C and 1 h) took 7 days for complete growth of mycelia. The mean mycelia growth rate (mm/day) of *Pleurotus* HK-37 followed the order: standard agar > treated agar of (30%; 2 h and 120°C) > treated agar of (30%; 2 h and 115°C) > treated agar of (20%; 2 h and 120°C) > treated agar of (10%; 0.5 h and 115°C) > nontreated agar (120°C and 1 h). The variations in colony diameter of *Pleurotus* HK-37 mycelium on six different PDA media are graphically shown in Figure 4. The mycelia of *Pleurotus* HK-37 obtained from low-cost agars grew well and fully colonized in both sterilized jars filled with sorghum grains (Figure 5). *Pleurotus* HK-37 mother spawns colonized after ten days of incubation.

## 4. Discussion

### 4.1. Effect of Different Variables of Agar on Growth of Pleurotus HK-37

This study assessed the interactive effect of varying gel strength of local agar on the growth rate of *Pleurotus* HK-37. As reported by the literature, gel strength stands as the most significant factor of agar quality. Agar quality of local *Gracilaria salicornia* is affected by extraction methods [22] and conditions used for extraction [6]. The independent variables (extraction temperature, alkali treatment duration, and NaOH concentration) affect the dependent variables (gel strength and sulfate content) of the local agar [6]. With the exception of gelling and melting temperatures, other variables appeared to affect the growth rate of *Pleurotus* HK-37. The results showed that the rate of growth of *Pleurotus* HK-37 varies between PDA media with different gel strength (Figure 4). Higher growth rate of *Pleurotus* HK-37 was seen in the PDA medium having agar with higher gel strength. Alkali concentration had significant positive correlation to gel strength of local agar; as a result, it affects the growth rate of *Pleurotus* HK-37. The relationship between gel strength and mycelia growth rate is highly significant (Table 2). The study has shown that the increasing firm of agar gel can provide enough support to the *Pleurotus* HK-37 mycelia to grow fast and comfortably in the media. In addition, alkali pretreatment increases syneresis property of agar which simplifies the removal of water from agar blocks during extraction process [23]. Higher water content in agar may contribute to the poor gelling ability resulting in softening of agar media, hence affecting the growth rate of *Pleurotus* HK-37.

The occurrence of lower gel strength in nontreated agar is attributed to the presence of high sulfate content [6]. As shown in Table 1, we found that the sulfate content of agar decreases as the alkali concentration increases during pretreatment, which indicates that alkali concentration plays a significant role in the process. Alkali pretreatment is responsible for the desulfation of agar and increase of gel strength [24, 25]. The sulfate content of agar showed significant relationship with mycelia growth rate of *Pleurotus* HK-37 at 0.05 confidence interval (Table 2) but with inversely proportion; higher sulfate content in agar resulted in lower growth rate of *Pleurotus* HK-37 in a medium.

### 4.2. Comparative Account on the Low-Cost Agar with the Commercially Available Agar

Comparative studies on tissue culture of *Pleurotus* HK-37 in PDA media gelled with local and bacteriological agars were done. In general, the stability and performance of local agars on active growth of *Pleurotus* HK-37 mycelia are quite comparable to bacteriological agar. However, the medium produced by nontreated agar was somewhat soft than the rest agar media. This could be explained by low gel strength of the agar. The same observation has been reported in [10]. All PDA plates appeared white and feathery and showed to grow in a circular mode (radial extension). The production of local agar was economically cheaper compared to the cost of imported agar. The extractions that yield 500 g of nontreated (120°C and 1 h) and treated (30%; 2 h and 120°C) agars cost about USD 38.9 and USD 116.4, respectively. This is cheaper compared to the cost of commercial agar which in Tanzania costs about USD 178.7 per 500 g in 2017. There is approximately 35–78% reduction in the cost of agar when extracted from local *Gracilaria salicornia*. It should be noted that this study was based on laboratory experiment which is usually fairly expensive. Industrial production might further lower the cost and scale-up agar production significantly by minimizing production time, ensuring high raw material input and high agar output. Both extracted agars show good performance in PDA media for active growth of *Pleurotus* HK-37 as it did on standard agar PDA. Thus, there is a need of using locally produced *Gracilaria salicornia* agar for tissue culture of *Pleurotus* HK-37 as it provides the comparable growth effect.

### 4.3. Efficiency and Suitability of Local Agar for Tissue Culture of *Pleurotus* HK-37

The efficiency and suitability of local agar from *Gracilaria salicornia* were evaluated by tissue culture of *Pleurotus* HK-37. *Pleurotus* HK-37 mycelia grow well in both tested local agars PDA media at ambient temperature of 27 ± 2°C for 5 to 7 days. These results are reliable with those of [1], as it used commercial agar on PDA media for culture of *Pleurotus* HK-37. Raymond et al. [26] also described the culture of *Pleurotus* HK-37 using malt extract agar (MEA) media; the results for growth rate of *Pleurotus* HK-37 were the same as those of this study. Moreover, this study shows that mycelia growth rate of *Pleurotus* HK-37 in PDA culture media is affected not only by substrates and nutrients which exist in media as reported by Mshandete [1] but also by the gel strength of agar. PDA plates with higher agar gel strength took shorter time to fully colonize the plates compared with the ones with lower gel strength. This is because higher gel strength of agar in media provides supreme gelling and thickening abilities (viscosity) which ensure maximum balance and movement of water and nutrients to spawns as reported in [27].

## 5. Conclusions

This study has shown the possibility to use local agar as a low-cost media for tissue culture of *Pleurotus* HK-37. The low-cost agar would be of great practical use to the mushroom cultivars as currently most of natives use local propagation methods because of the media cost. This innovated agar might lessen the challenges facing the adoption and utilization of *Pleurotus* HK-37 species and also reduce the dependence of importing agar to Tanzania. The present study used conventional method for agar extraction which slightly is of energy- and solvent-consuming. A quantity of sodium hydroxide (NaOH) was used for pretreatment of *Gracilaria salicornia* at an elevated temperature for several hours; this generates solution wastes such as alkaline residues and sodium agaropectinates. Reasonably, for future studies, a new extraction technique based on microwave-assisted extraction (MAE) should be tested for production cost of agar from local *Gracilaria salicornia* as this process has been reported to require less energy and solvents and is also eco-friendly as it generates fewer wastes [28, 29]. Despite the use of conventional method, this process of extraction is suitable for the scale-up of low-cost agar. The chosen experimental design approaches on simple route that limits the use of high-cost hazardous chemicals and high usage of energy. The chemicals used are not of potential hazardous group, and the reaction parameters and conditions are controllable. As not enough, the free-cost raw material (*Gracilaria salicornia*) used ensures maximum input with minimum waste output. This reliable and well-defined extraction process may minimize scale-up difficulties and hence could increase the batch size in a full-scale production plant.

## Figures and Tables

**Table 1:** Physicochemical characteristics of 1.5% (w/v) agar (local and standard), production cost, and mycelia growth rate (mm/day) of *Pleurotus* HK-37 on PDA media at 27 ± 2°C (mean ± SD, *n* = 3).

SN	Form	Agar	Gel strength (g/cm^2^)	Sulfate content (%)	Gelling temp. (°C)	Melting temp. (°C)	Cost of agar per 500 g in USD^*∗*^	Mycelia growth rate (mm/day) of *Pleurotus* HK-37
1	Nontreated	Agar extracted at 120°C and 1 h	159.0 ± 2.5	2.14 ± 0.04	39.7	89.0	38.9	9.87 ± 1.44

2	Treated	Agar extracted at 10%, 0.5 h and 115°C	282.1 ± 5.4	0.76 ± 0.13	41.7	86.9	77.5	11.2 ± 1.15
3		Agar extracted at 20%, 2 h and 120°C	398.0 ± 10	0.43 ± 0.03	39.7	85.1	95.6	12.4 ± 1.50
4		Agar extracted at 30%, 2 h and 115°C	407.3 ± 9.3	0.32 ± 0.04	40.0	88.1	109.8	13.1 ± 1.60
5		Agar extracted at 30%, 2 h and 120°C	510.3 ± 16	0.29 ± 0.04	39.3	88.4	116.4	14.9 ± 0.85

6	Standard	Agar powder, bacteriological	613.8 ± 10	0.26 ± 0.02	36.0	85.4	178.7	15.4 ± 0.98

^*∗*^Equivalent cost in USD; the currency conversion rate as per 2017: 1 USD = 2240 TSh (Tanzanian shilling).

**Table 2:** Correlations and interactions of variables of local agar on the growth of Pleurotus HK-37 using Pearson product-moment correlation coefficient.

Parameter	NaOH concentration (%)	Sulfate content (%)	Gel strength (g/cm^2^)	Gelling temp. (°C)	Melting temp. (°C)	Mycelia growth rate (mm/day)
NaOH concentration (%)	1					
Sulfate content (%)	−0.897^*∗*^	1				
Gel strength (g/cm^2^)	0.953^*∗∗*^	−0.908^*∗*^	1			
Gelling temp. (°C)	−0.350	−0.010	−0.370	1		
Melting temp. (°C)	−0.110	−0.450	−0.220	−0.24	1	
Mycelia growth rate (mm/day)	0.939^*∗∗*^	−0.827^*∗*^	0.979^*∗∗*^	−0.43	−0.02	1

^*∗*^Correlation is significant at the 0.05 level (one-tailed). ^*∗∗*^Correlation is significant at the 0.01 level (one-tailed).

## Data Availability

The data used to support the findings of this study are included within the article.
